# Article title efficacy and safety of romosozumab in postmenopausal women with osteoporosis previously treated with antiresorptive drugs: a prospective observational study and literature review

**DOI:** 10.3389/fgwh.2026.1779730

**Published:** 2026-07-02

**Authors:** Misa Yamamoto, Yuka Ikenaga, Gaku Yamamoto, Tadashi Oride, Yukako Oi, Airi Kuruma, Michiko Bun, Taro Yagi, Aasa Shimizu, Yasuto Kinose, Mahiru Kawano, Michiko Kodama, Kenjiro Sawada

**Affiliations:** 1 Department of Obstetrics and Gynecology, Graduate School of Medicine, The University of Osaka, Suita, Osaka, Japan; 2 Department of Obstetrics and Gynecology, Kansai Rosai Hospital, Amagasaki, Hyogo, Japan; 3 Department of Obstetrics and Gynecology, Itami City Hospital, Itami, Hyogo, Japan; 4 Department of Obstetrics and Gynecology, Aizenbashi Hospital, Osaka, Osaka, Japan; 5 Department of Obstetrics and Gynecology, Rinku General Medical Center, Izumisano, Osaka, Japan; 6 Department of Obstetrics and Gynecology, Suita Municipal Hospital, Suita, Osaka, Japan; 7 Department of Obstetrics and Gynecology, Osaka General Medical Center, Osaka, Osaka, Japan; 8 Department of Obstetrics and Gynecology, Sakai City Medical Center, Sakai, Osaka, Japan; 9 Department of Obstetrics and Gynecology, Ehime University Graduate School of Medicine, Toon, Japan

**Keywords:** bisphosphonate, denosumab, postmenopausal osteoporosis, romosozumab, switching therapy

## Abstract

**Objectives:**

The number of patients receiving long-term antiresorptive drugs for postmenopausal osteoporosis is increasing worldwide. However, there is limited evidence on when and how to discontinue or switch these drugs. This study evaluated the efficacy of romosozumab in women who had been treated with antiresorptive drugs for over three years.

**Methods:**

This was a prospective observational non-randomized study analyzing patients with postmenopausal osteoporosis, which was registered in the jRCT (No. 1051210070). Postmenopausal women with lumbar and femoral neck bone mineral density (BMD) below −2.5 SD, as measured by dual-energy x-ray absorptiometry (DXA), despite more than 36 months of antiresorptive treatment, were recruited. They received romosozumab along with calcium and active vitamin D supplementation for 12 months. BMD and bone turnover markers, including serum P1NP and I-CTP, were periodically monitored.

**Results:**

Twenty-one patients (median age: 73 years) were observed for a median of 833 days. Baseline BMD was 0.77 g/cm² at the lumbar spine (T-score: −2.0) and 0.49 g/cm² at the femoral neck (T-score: −2.8). After 12 months, lumbar BMD significantly increased by 6.2% (*P* < 0.001), whereas femoral neck BMD remained unchanged (*P* = 0.46). P1NP and I-CTP levels significantly increased by 455.8% and 30.3%, respectively (*P* < 0.001). One patient developed periodontitis and a vertebral fracture. Multiple regression analysis identified baseline BMD and P1NP values as significant predictors of lumbar BMD changes.

**Conclusion:**

Romosozumab significantly increased lumbar BMD and bone turnover markers in patients previously treated with antiresorptive drugs, supporting its use as a potential treatment option.

**Clinical Trial Registration:**

https://jrct.mhlw.go.jp/, identifier No. 1051210070.

## 1 Introduction

In developed countries, the number of patients with osteoporosis is increasing owing to the aging of population. Japan is leading in life expectancy worldwide, with 87 years for women and 81 years for men (1). According to the Research on Osteoarthritis/Osteoporosis Against Disability study in 2005, there were 6.4 and 17.3 million patients with lumbar spine and hip osteoporosis, respectively, in Japan (2). The bone mineral density (BMD) declines with age in the entire population; however, women are at a higher risk because of rapid bone loss around menopause. Postmenopausal osteoporosis is a common bone disease and a serious condition that makes women twice as likely as men to have bone fractures. It is associated with high medical costs and reduced quality of life and life expectancy (3). As osteoporosis is not considered fatal in itself, people with osteoporosis can expect to live 15 years or more after diagnosis, highlighting the importance of developing treatment strategies for long-term management (4).

Two types of osteoporosis drugs exist: antiresorptive and anabolic agents. Antiresorptive drugs include bisphosphonates (e.g., alendronate, etidronate, risedronate, pamidronate, and zoledronate), selective estrogen-receptor modulators (raloxifene and bazedoxifene), active vitamin D3 derivatives (alfacalcidol and eldecalcitol), a fully human monoclonal antibody to receptor activator of nuclear factor *κ*-B ligand (RANKL; denosumab), and thyroid hormone (calcitonin). Anabolic agents include parathyroid hormone (teriparatide), parathyroid-related peptide synthetic analogs (abaloparatide), and sclerostin inhibitors (romosozumab) (5). Among these treatment options, romosozumab is an antisclerostin antibody that inhibits sclerostin. Sclerostin is a glycoprotein that decreases osteoblast-mediated bone formation and increases osteoclast-mediated bone resorption. Thus, romosozumab has a strong dual effect on the enhancement of bone formation and the inhibition of bone resorption, resulting in improved bone strength in patients with osteoporosis (6). Romosozumab has been shown to significantly improve BMD by 13% in the lumbar spine and 5% in the femoral neck compared to placebo as a new treatment (7). It was covered by the health insurance in Japan in 2019.

With the extension of life expectancy, the number of patients receiving long-term antiresorptive osteoporosis drug therapy (usually >5 years) is increasing; however, a certain number of patients discontinue these drugs after 5 years of therapy or earlier. An important reason for the discontinuation of antiresorptive drugs is the fear of adverse effects, such as atypical femoral fracture (AFF) and osteonecrosis of the jaw (ONJ), which may occur, especially in long-term antiresorptive drug users (8). However, there is little evidence to guide decisions regarding how to stop or switch antiresorptive drugs. The task force of the American Society for Bone and Mineral Research suggested that after 3–5 years of bisphosphonate treatment, a drug holiday of 2–3 years can be considered for women not at high fracture risks. However, in high-risk women, the continuation of treatment for up to 10 (oral) or 6 years (intravenous) should be considered because the incidences of AFF and ONJ are rare, and such rare events should not be outweighed by vertebral fracture risk reduction in high-risk patients (9).

Among the candidate drugs for switching treatments, romosozumab, which not only promotes bone formation but also inhibits bone resorption, appears to be ideal for patients previously treated with antiresorptive drugs. However, clinical evidence for the use of romosozumab, which has been switched from conventional osteoporosis drugs, remain lacking, and further data are needed. Thus, we aimed to investigate the efficacy of romosozumab in women who had been treated with conventional antiresorptive osteoporosis drugs for more than 3 years.

## 2 Material and methods

### 2.1 Patients and study design

**Figure 1:**
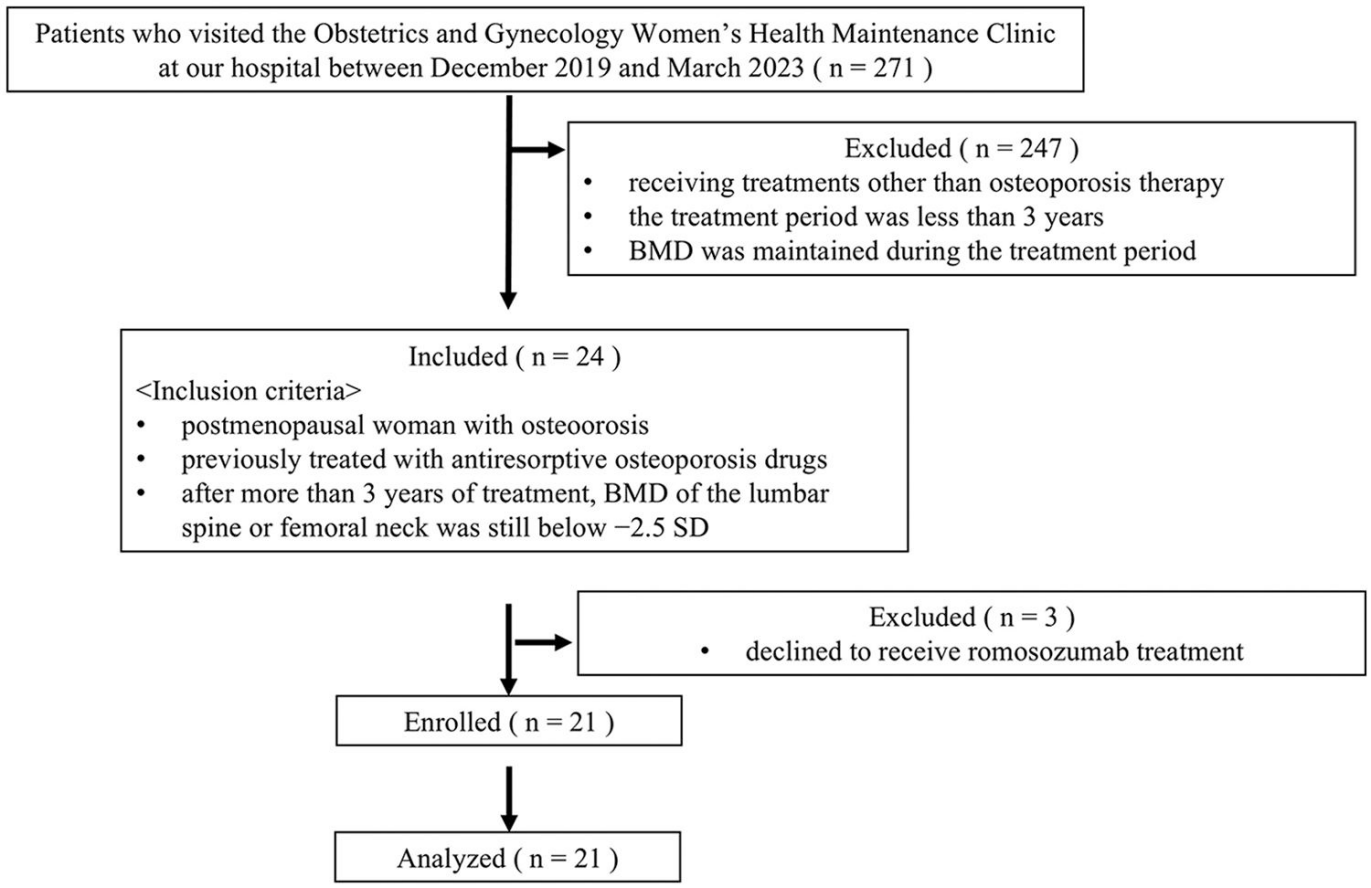
STROBE-style flowchart of this study.

**Figure 2:**
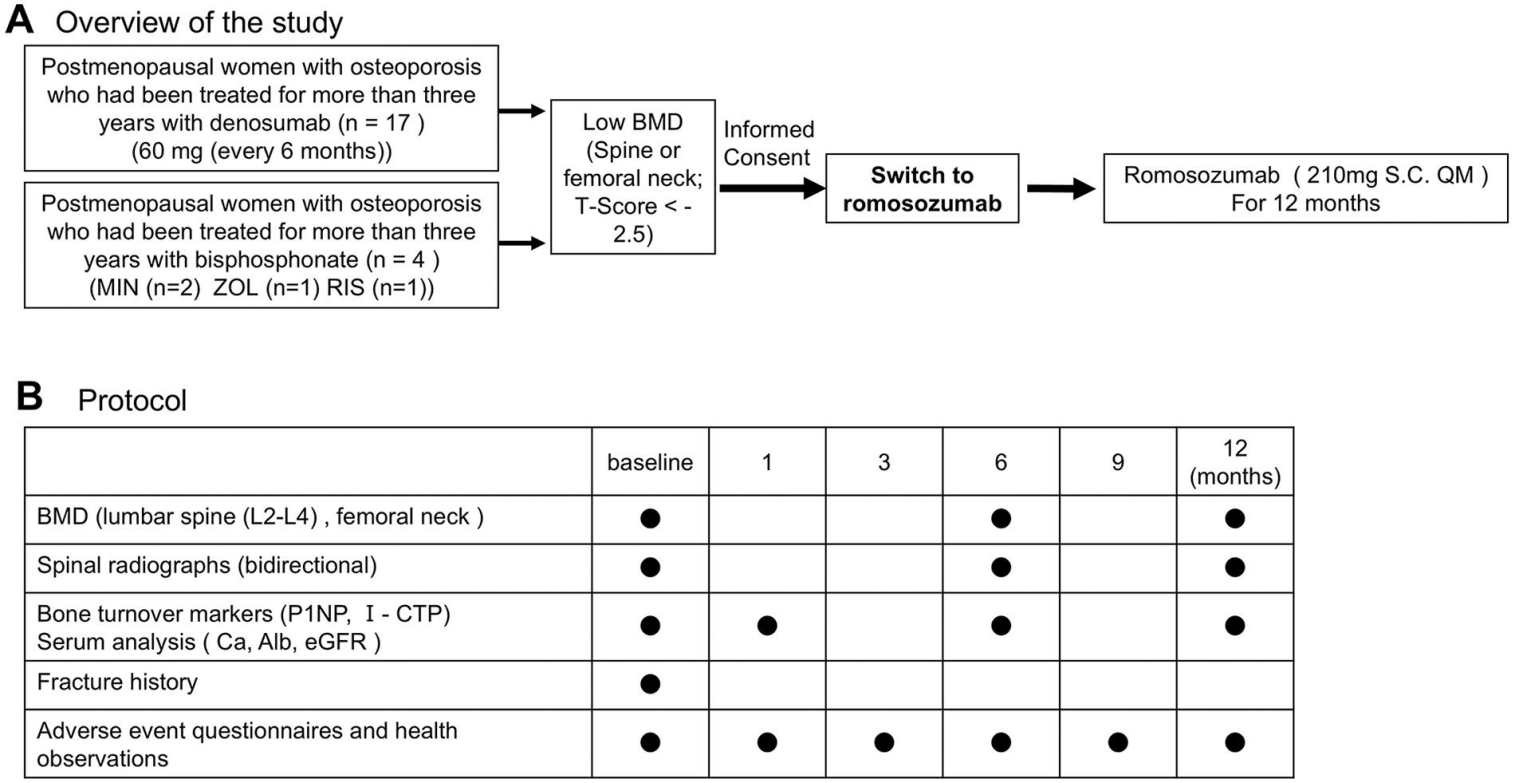
Overview of the study **(A)** and protocol **(B);** MIN, minodronate; ZOL, zoledronate; RIS, risedronate; PINP, type I collagen N-terminal propeptide; I-CTP, type I collagen cross-linked C-telopeptide.

This was a prospective observational non-randomized study analyzing patients with postmenopausal osteoporosis between December 2019 and March 2023. It was conducted in accordance with the ethical standards of the Helsinki II Declaration and approved by Osaka University Clinical Research Review Committee (approval number: 19043). This study was registered in the jRCT (No. 1051210070) on 24/08/2021. The written informed consent was obtained from the participants. Figure 1 presents the STROBE-style flowchart of this study. At our hospital, 271 patients visited the Obstetrics and Gynecology Women's Health Maintenance Clinic between December 2019 and March 2023. Of these, 21 patients met the eligibility criteria and were included in the analysis. Figure 2 shows an overview of the study (Figure 2A) and protocol (Figure 2B). All participants in this study were Japanese women aged 37–85 years. The patients were naturally or surgically menopausal (>12 months after the last menstrual period or after an oophorectomy). The BMDs of the lumbar region (at the L2–L4 vertebral site) and femoral neck were measured using dual-energy x-ray absorptiometry (DXA) (until Oct 2020: Discovery-A, Hologic, Bedford, MA, USA, and since Nov 2020: Horizon, TOYO Medic, Tokyo, Japan). At our institution, the coefficient of variance is routinely monitored and confirmed as <1.0% for the lumbar spine. The patients who were diagnosed with osteoporosis (below −2.5 SD of the mean value for healthy Japanese 20–44-year-old women) were initially treated with antiresorptive osteoporosis drugs, including alendronate, risedronate, minodronate, and denosumab. After more than 3 years of treatment, the patients whose BMD of the lumbar spine or femoral neck was still below −2.5 SD of the mean value for healthy Japanese 20–44-year-old women, were recruited to participate in this study. The exclusion criteria were: 1) history of hypersensitivity to any component of sclerostin, 2) hypocalcemia, and 3) patients who were otherwise deemed unsuitable for the study by the treating physician (e.g., patients with cardiovascular or cerebrovascular disease events in the year before starting romosozumab therapy). The participants who provided written informed consent received 12 months of treatment with romosozumab (Evenity, Amgen Inc., Thousand Oaks, CA). Romosozumab (210 mg) was administered via subcutaneous injection once monthly for 12 months. All patients received daily calcium (600 mg) and Alfacalcidol (active vitamin D3 analog; 1 µg) during the study period.

The primary endpoint was the percentage change in BMD at the lumbar spine and femoral neck after 6 or 12 months of romosozumab treatment. The secondary endpoints were the percentage change in bone metabolism markers [bone formation marker type I procollagen-N-propeptide (P1NP) and bone resorption marker type I collagen cross-linked C-telopeptide (I-CTP)] after 6 or 12 months of romosozumab treatment. Adverse events were also recorded during the follow-up period.

### 2.2 Study assessments

The DXA scans were obtained at baseline, and 6, and 12 months after romosozumab administration on the posteroanterior projections of the lumbar spine (L2–L4) and femoral neck. Serum analysis was performed at baseline, and 1, 6, and 12 months after romosozumab administration; P1NP [SRL (Tokyo, Japan)] (range: 16.8–70.1 ng/mL for premenopausal women; 26.4–98.2 ng/mL for postmenopausal women) was measured as a bone formation marker and I-CTP (SRL) (range: <4.5 ng/mL) was measured as a bone resorption marker. In addition, to observe the adverse events, serum calcium (Ca), serum albumin (Alb), liver function (AST and ALT), renal function [urinalysis, serum creatinine (sCr), and blood urea nitrogen], lipid profile (total cholesterol, HDL-cholesterol, triglycerides, and LDL-cholesterol), and fasting blood glucose were measured. The estimated glomerular filtration rate (eGFR) was calculated using the following formula: eGFR (mL/min/1.73 m^2^) = 194 × sCr (mg/dL)^−1.094^ × age^−0.287^ × 0.739, (if female) as previously determined (10). Spinal radiographs (bidirectional) were obtained at baseline, and 6, and 12 months after romosozumab administration to evaluate the presence of fractures. The adverse event questionnaires and health observations were administered at baseline, and 1, 3, 6, 9, and 12 months after romosozumab administration.

### 2.3 Statistical analysis

The percentage changes from the baseline in BMD, P1NP, and I-CTP were presented as means and 95% confidence intervals (CI). A paired student's t-test was performed to compare the BMD and bone metabolism markers. Each parameter was compared between groups using a non-parametric Wilcoxon signed-rank test. The results are expressed as mean ± standard error; *P*-value of 0.05 or less was considered statistically significant. All statistical analyses were performed using EZR (Saitama Medical Center, Jichi Medical University, Saitama, Japan), which is a graphical user interface for R version 4.3.2 (The R Foundation for Statistical Computing, Vienna, Austria) (11).

The planned sample size was set at 20 participants. The rationale was as follows. The primary endpoint was defined as an increase in the rate of change in lumbar spine and femoral neck BMD from baseline that exceeded the minimum clinically meaningful change. Based on multiple previous clinical trials, the efficacy threshold was set at 1%. Given that the lower of the two BMD change rates (lumbar spine or proximal femoral neck) is approximately 3%, the number of participants required to demonstrate that the observed BMD change is statistically significantly greater than 1% was calculated to be 15, assuming a two-sided significance level of 5% and a power of 80%. Allowing for potential dropouts, the target sample size was set at 20. No *post hoc* power analysis was performed.

## 3 Results

### 3.1 Participants

Table 1 shows the clinical characteristics of patients. A total of 21 patients, aged 37–85 (median: 73) years were enrolled in this study, and 17 patients (81%) were older than 65 years. The median BMI of all patients was 19.1 (range: 14.8–23.2) kg/m^2^. The median observation period was 833 (range: 378–1,141) days; 17 patients (81.0%) received denosumab and four patients received bisphosphonates (2; minoderonate, 1; zoledronate, 1: risedronate) prior to romosozumab administration. The median duration of previous treatment was 58.5 (range: 36–88) months in the denosumab group and 68 (range: 46–91) months in the bisphosphonate group. The median time from menopause onset to romosozumab administration was 23 (range: 8–37) years. The median BMD before the start of romosozumab treatment was 0.77 g/cm^2^ (−2.1 SD) in the spine (range: 0.57–1.16) and 0.49 g/cm^2^ (−2.8 SD) in the femoral neck (range: 0.38–0.55). Five patients (23.8%) experienced fragility fractures prior to romosozumab administration. The median corrected serum Ca was 9.9 (range: 9.1–10.4) mg/dL, the median eGFR was 65.7 mL/min/1.73 m^2^ (range: 45.9–138.6), and no patient had serious hepatic or renal dysfunction prior to romosozumab administration.

**Table 1:** Baseline characteristics.

	All (*n* = 21)	Denosumab (*n* = 17)	Bisphosphonates (*n* = 4)
Variables	median (range), *; *n* (%)	median (range), *; *n* (%)	median (range), *; *n* (%)
Age (years)	73.0 (37–85)	74.5 (52–85)	69.0 (56–82)
Height (cm)	153.0 (143–165)	151.5 (143–161)	154.5 (153–165)
Weight (kg)	43.0 (31–62)	43.0 (31–58)	46.0 (39–62)
BMI (kg/m^2^)	19.1 (14.7–23.2)	19.2 (14.7–23.2)	19.2 (16.4–22.8)
Age at menopause (years)	49.5 (35–52)	50 (35–52)	46 (46–48)
Number of cases with surgical menopause	2	1	1
Previous vertebral fracture	*5 (23.8%)	*5 (29.4%)	*0 (0%)
Previous nonvertebral fracture	*1 (4.8%)	*1 (5.9%)	*0 (0%)
Pretreatment denosumab 60 mg (every 6 months)	*17 (81.0%)	*17 (100%)	
Bisphosphonates: minodronate 50 mg (monthly p.o.)	*2 (9.5%)		*2 (50%)
: zoledronate 5 mg (yearly i.v.)	*1 (4.8%)		*1 (25%)
: risedronate 75 mg (monthly p.o.)	*1 (4.8%)		*1 (25%)
Duration of treatment for immediate pretreatment (months)	60 (36–91)	58.5 (36–88)	68 (46–91)
Interval from final prior treatment prescription (months)	6 (1–16)	6 (6–11)	3.5 (1–16)
Lumbar BMD (g/cm^2^)	0.77 (0.571–1.16)	0.78 (0.571–1.16)	0.76 (0.715–0.88)
Lumbar BMD (T-score)	−2.0 (−3.6–1.1)	−1.9 (−3.6–1.3)	−2.1 (−2.5–−1.1)
Femoral neck BMD (g/cm^2^)	0.49 (0.375–0.551)	0.50 (0.378–0.551)	0.45 (0.375–0.546)
Femoral neck BMD (T-score)	−2.8 (−4.1–−1.5)	−2.7 (−4.1–−1.5)	−3.4 (−3.8–−2.7)
Serum P1NP (*μ*g/L)	15.7 (8.4–244)	15.3 (8.4–244)	21.2 (10.9–27.0)
Serum I-CTP (ng/mL)	3.2 (1.9–11.3)	3.2 (1.9–11.3)	3.5 (3.2–3.8)
Corrected serum Ca (mg/dL)	9.9 (9.1–10.4)	9.8 (9.4–10.4)	9.8 (9.1–10.4)
eGFR (mL/min/1.73 m^2^)	64.6 (34.4–138.6)	61.9 (34.4–138.6)	67.1 (51.7–94)

BMD, bone mineral density; P1NP, procollagen type 1 N-terminal propeptide; I-CTP, type I collagen cross-linked C-telopeptide; p.o., oral administration; IV, intravenous; eGFR, estimated Glomerular Filtration Rate.

*means the number of participants and this explanation is included in Figure legends.

### 3.2 Changes in BMD

**Figure 3:**
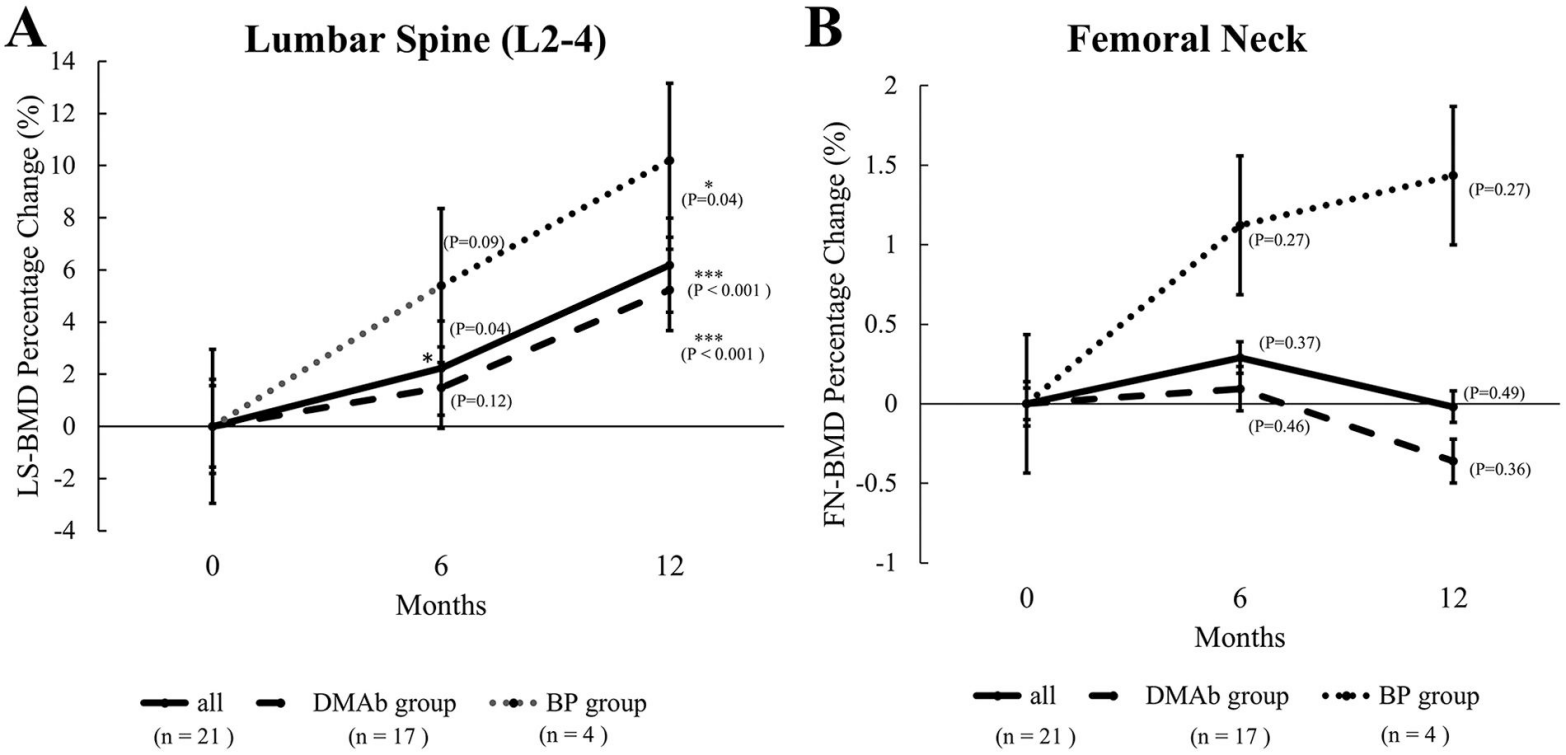
Percent changes from the baseline in bone mineral density at lumbar spine **(A)** and femoral neck **(B)** during romosozumab treatment. The thick lines indicate total data. The dashed and dotted lines represent percent changes from the baseline for patients who had received denosumab and bisphosphonates, respectively. BP, bisphosphonate; DMAb, denosumab. Bars indicate mean ± standard errors. *P < 0.05, ***P < 0.001; change from the baseline in each treatment group.

The percent change in lumbar BMD from baseline is shown in Figure 3A. There was a significant increase by 2.2% (*P* < 0.05) at 6 months from the baseline and 6.1% (*P* < 0.001) at 12 months of romosozumab treatment. In patients treated with denosumab before romosozumab administration, the rate increased by 1.5% (*P* = 0.12) at 6 months and 5.2% (*P* < 0.001) at 12 months, and in those who had received bisphosphonates, the rate increased by 5.4% (*P* = 0.09) at 6 months and 10.2% (*P* < 0.05) at 12 months.

The percentage change in femoral neck BMD from baseline is shown in Figure 3B. These values did not differ significantly and remained unchanged before and after romosozumab treatment (*P* = 0.49). The percent of change in BMD was not significantly different from baseline to 12 months for those treated with denosumab (−0.4%) and bisphosphonates (1.4%) before romosozumab treatment.

### 3.3 Bone turnover markers

**Figure 4:**
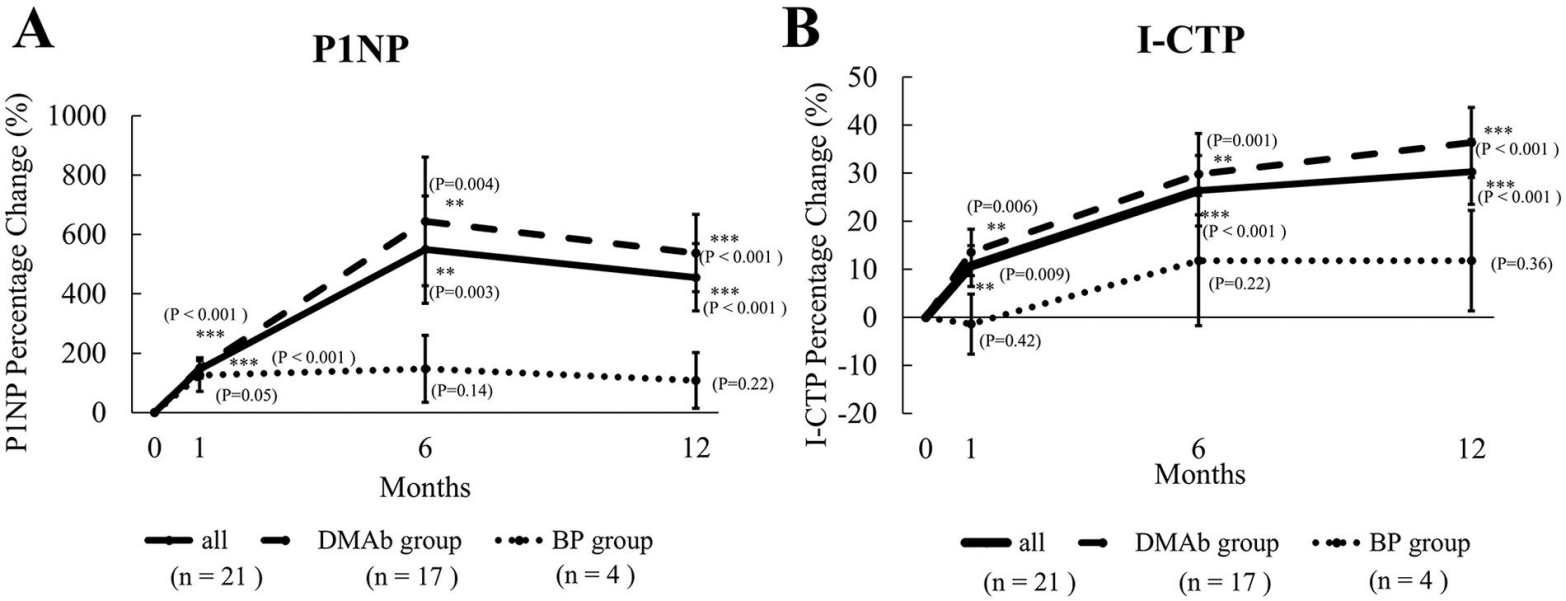
Percent changes from the baseline in serum P1NP **(A)** and I-CTP **(B)** during romosozumab treatment. The thick lines indicate total data. The dashed and dotted lines represent percent changes from the baseline for patients who had received denosumab and bisphosphonates, respectively. BP, bisphosphonate; DMAb, denosumab; P1NP, procollagen type 1 N-terminal propeptide; I-CTP, type I collagen cross-linked C-telopeptide. Bars indicate mean ± standard errors. **P < 0.01, ***P < 0.001; change from the baseline in each treatment group.

Figure 4 shows the percent changes from baseline serum P1NP (Figure 4A) and I-CTP levels (Figure 4B). P1NP increased significantly from baseline to 145.8%, 549.2%, and 455.8% after 1 (*P* < 0.001), 6 (*P* < 0.01), and 12 (*P* < 0.001) months, respectively, of romosozumab treatment. The values in the denosumab group significantly increased to 537.5% at 12 months (*P* < 0.001), while the values in the bisphosphonate group slightly increased to 108.9% at 12 months; however, the difference was not significant (*P* = 0.16). I-CTP increased significantly from baseline to 10.7%, 26.4%, and 30.3% after 1, (*P* < 0.01), 6 (*P* < 0.001), and 12 months (*P* < 0.001), respectively, of romosozumab treatment. The values in the denosumab group significantly increased to 36.4% at 12 months (*P* < 0.001), while the values in the bisphosphonate group increased to 4.2% at 12 months; however, the difference was not significant (*P* = 0.36).

### 3.4 Factors affecting the changes in the rate of BMD by romosozumab administration

To investigate the predictors influencing the change in each BMD value at 12 months, multiple regression analyses of various confounding factors (BMD before and 6 months after romosozumab administration, type of treatment before romosozumab administration, and P1NP or I-CTP values at 0, 1, 6, and 12 months) were performed (Supplementary Table S1). The regression coefficient analyses showed that factors affecting the change in the rate of lumbar BMD at 12 months were the baseline spine BMD T-score before romosozumab (partial regression coefficient = −1.39, *P* < 0.001) and baseline P1NP values (partial regression coefficient = 0.00015, *P* < 0.05). In contrast, the baseline femoral neck BMD T-score before romosozumab administration affected the rate of change in femoral neck BMD at 12 months (partial regression coefficient = −2.1, *P* < 0.001) (Supplementary Table S2).

### 3.5 Safety and adverse events

All 21 patients completed the 12 months of romosozumab administration. After treatment, the patients were switched to bone resorption inhibitors, and osteoporosis treatment was continued. Seventeen patients resumed treatment with denosumab, two with minodronate, and one with risedronate; one patient had not yet resumed treatment with bone resorption inhibitors because of dental treatment. No serious adverse events were observed; one patient (4.8%) had periodontitis and a new vertebral fracture. The patient who had been pretreated with denosumab had a history of compression fracture of the thoracic spine prior to treatment and developed mild periodontitis after the first dose of romosozumab. Three months later, a thoracic spine radiograph revealed a new compression fracture of the thoracic spine, which resolved mildly with non-obstructive treatment, and the patient subsequently completed the 12 doses of romosozumab treatment without any problem. No other patients experienced new fractures after receiving romosozumab. None of the patients had symptoms of suspected cardiovascular or cerebrovascular disease and no deaths were associated with romosozumab treatment.

The mean values of corrected serum Ca and eGFR during romosozumab treatment are shown in Supplementary Figure S1. The mean values of corrected serum Ca were 9.9, 9.8, 9.9, and 9.9 mg/dL before the start, and after 1, 6, and 12 months, respectively, of romosozumab treatment. No patient exhibited symptoms of hypocalcemia. The mean values of eGFR were 66.4, 84.5, 67.0, and 68.3 mL/min/1.73 m^2^ before the start, and after 1, 6, and 12 months, respectively, of romosozumab treatment. No patient developed severe renal dysfunction.

## 4 Discussion

Herein, we evaluated the efficacy and safety of romosozumab in women who were treated with conventional antiresorptive osteoporosis drugs, such as bisphosphonates and denosumab for more than 3 years, and found that 12 months of romosozumab administration significantly increased lumbar BMD by 6.1% although it did not significantly affect femoral neck BMD. Regarding bone turnover markers, P1NP significantly increased by 455.8% and I-CTP significantly increased by 30.3% at 12 months after romosozumab administration. No severe adverse events were reported. Thus, we concluded that romosozumab was effective in postmenopausal osteoporosis patients who were treated with antiresorptive agents such as bisphosphonates and denosumab for a long time. In a multinational FRAME phase 3 trial of romosozumab in postmenopausal women with osteoporosis, romosozumab as an initial therapy increased lumbar BMD by 13.3% and femoral neck BMD by 5.2% after 12 months.^12^ In this study, the increase in lumbar BMD was 6.2% and no significant differences were observed in femoral neck BMD; both the values were lower than those in the FRAME study. These differences may have been caused by prior treatments with bisphosphonates and denosumab.

In this study, lumbar BMD of the patients who had received denosumab prior to romosozumab administration significantly increased by 5.2% at 12 months, whereas femoral neck BMD did not increase significantly. Several studies have demonstrated the therapeutic effects of switching from denosumab to romosozumab (Table 2). McClung MR et al. reported that switching from 12 months of denosumab treatment to romosozumab (*n* = 16) increased lumbar BMD by 5.3% and femoral neck BMD by 1.0% after 12 months (12). Ebina et al. reported that switching from denosumab treatment for a mean of 24.1 months to romosozumab (*n* = 45) increased lumbar BMD by 6.4% (*P* < 0.001) and femoral neck BMD by 1.5% (NS) after 12 months (13). Tominaga et al. reported an increase in lumbar BMD by approximately 8.5% with romosozumab after denosumab treatment; however, femoral neck BMD did not increase significantly after 12 months (*n* = 46) (14). In a Phase 2 study of patients who received 24 months of romosozumab followed by 12 months of denosumab, followed by a second dose of romosozumab for 12 months, the second dose of romosozumab increased the spinal BMD by 2.3% and femoral neck BMD by 0.8% (*n* = 16) (15), which was similar to the present study. Compared to these studies, this study had a longer duration of denosumab pretreatment (median, 57 months; range 36–78 months), indicating that romosozumab can increase lumbar BMD even after prolonged denosumab treatment. Regarding bone turnover markers, in the phase III FRAME study, the bone formation marker P1NP increased quickly after the romososumab administration initiation and the bone resorption marker *β*-CTX decreased quickly after the treatment initiation and then returned to its original level (7). In contrast, when patients were switched from denosumab to romosozumab, bone formation markers increased over 6–12 months to approximately 100%–300% and bone resorption markers continued to increase over 12 months to approximately 80%–200% in previous reports (12–15). In this study, similar to previous reports, the bone formation marker P1NP increased to 643.8% at 6 months and 537.4% at 12 months after romosozumab administration, whereas the bone resorption marker I-CTP continued to increase to 364% at 12 months. These data suggest that denosumab reversed the inhibitory effect on bone resorption and enhanced the bone metabolic turnover, leading to increased BMD.

**Table 2:** Effects of romosozumab treatment when switching from denosumab to romosozumab.

Author, Year	*n*	Duration of prior treatment (months) mean, SD	Duration of romosozumab treatment (months)	Percent Change in lumbar spine BMD (%) mean, SD	Percent Change in femoral neck BMD (%) mean, SD	Percent Change in total hip BMD (%) mean, SD	Bone fracture *n* (%)	Percent Change of bone formation marker	Percent Change of bone resorption marker	Adverse effect *n* (%)
Ebina et al. (2021) (13)	45	24.1 ± 15.8	12	6.4 ± 0.6 (*P* < 0.001)	0.7 ± 0.8 (NS)	0.6 ± 0.9 (NS)	3 (6.7%)	P1NP; 250.0%*	TRACP-5b; 80.0%*	NR
Tominaga et al. (2021) (14)	46	NR	12	8.5*	NR	3.8* NS	NR	iP1NP; 222.9%	TRACP-5b; 224.6%	No serious
McClung et al. (2021) (12)	16	Placebo 24.0 →denosumab 12.0	12	5.3 (3.2, 7.4) mean (95% CI)	1.0 (−1.0, 2.9) mean (95% CI)	0.9 (−0.1, 1.8) mean (95% CI)	0	P1NP; 100.0%*	*β*CTX; 75.0%*	1 (6.7%) (Serious)
Kendler et al. (2019) (15)	16	Romosozumab 24.0 →denosumab 12.0d	12	2.3 (0.3, 4.4) mean (95% CI)	0.8 (−0.3, 2.0) mean (95% CI)	−0.1 (−1.2, 0.9) mean (95% CI)	0	P1NP; 100.0%*	βCTX; 75.0%*	NS
This study	17	54.4 ± 11.2	12	5.2 ± 0.05 (*P* < 0.001)	−0.4 ± 0.04 (NS)	−4.5 ± 0.05 (NS)	1 (5.9%)	P1NP; 537.4% (*P* < 0.001)	I CTP; 36.4% (*P* < 0.0001)	No serious

BMD, bone mineral density; iP1NP, intact type I procollagen N-terminal propeptide; I-CTP, type I collagen cross-linked C-telopeptide; TRAP-5b, isoform 5b of tartrate-resistant acid phosphatase; β-CTX, β-isomer of C-terminal telopeptide of type 1 collagen; NR, not reported; NS, not significant.

* Approximate values inferred from the graphs.

In contrast, the lumbar BMD of the patients who had received bisphosphonates prior to romosozumab administration significantly increased by 10.2% at 12 months, while the femoral neck BMD increased by 1.4% at 12 months. Several studies on therapeutic effects of switching from bisphosphonates to romosozumab are summarized in Table 3. Ebina et al. reported that switching from bisphosphonate therapy for a mean of 28.1 months to romosozumab (*n* = 37) increased the spinal BMD by a mean of 10.2% (*P* < 0.001) and femoral neck BMD by 3.1% (*P* < 0.01) at 12 months (13). Shimizu et al. reported that romosozumab treatment (*n* = 38) after an average of 26.2 months of bisphosphonate treatment increased the lumbar BMD by approximately 12% and femoral neck BMD by approximately 3% at 12 months (16). Tominaga et al. reported that romosozumab administration after bisphosphonate treatment increased the lumbar BMD by approximately 8%, whereas no significant increase in total hip BMD were seen at 12 months (*n* = 54) (14). Inage et al. reported that switching from bisphosphonate treatment to romosozumab at 22.6 ± 14.9 months (*n* = 35) increased the lumbar BMD by 4.5% and femoral neck BMD by 1.0% after 6 months (17). Furthermore, in a phase III STRUCURE trial, romosozumab treatment (*n* = 206) after a mean of 6.2 years of bisphosphonate therapy increased the lumbar BMD by 9.8% and femoral neck BMD by 3.2% at 12 months (18). The results of this study are similar to those of previous reports, with a significant increase in lumbar BMD. Compared to previous studies, a longer duration of bisphosphonate treatment was used [68 (range: 46–91) months], suggesting that lumbar BMD significantly increased even after long-term bisphosphonate therapy. Regarding bone metabolism markers, previous reports have suggested that bone formation markers increased by approximately 20%–80% at 12 months, while bone resorption markers showed a suppressive trend between −10% and 20% (13, 14, 16–18). Similarly, in this study, the bone formation marker P1NP increased by 147.3% at 6 months and 108.9% at 12 months after romosozumab administration, whereas the bone resorption marker-CTP increased by 4.2% at 12 months, which was not significant. Our findings indicate that switching from bisphosphonates to romosozumab yields more favorable and predictable BMD responses than switching from denosumab to romosozumab as previously reported (13, 14). This difference may be influenced by the rebound phenomenon that occurs after denosumab discontinuation, characterized by a marked surge in bone turnover, rapid BMD loss, and an increased risk of multiple vertebral fractures (19). High-squality randomized studies directly comparing these treatment sequences are currently lacking and represent an important unmet clinical need.

**Table 3:** Effects of romosozumab treatment when switching from bisphosphonates to romosozumab.

Author, Year	*n*	Duration of prior treatment (months) mean, SD	Duration of romosozumab treatment (months)	Percent Change in lumbar spine BMD (%) mean, SD	Percent Change in femoral neck BMD (%) mean, SD	Percent Change in total hip BMD (%) mean, SD	Bone fracture *n* (%)	Percent Change of bone formation marker mean, SD	Percent Change of bone resorption marker mean, SD	Adverse effect *n* (%)
Ebina et al. (2021) (13)	37	28.1 ± 23.3	12	10.2 ± 0.9 (*P* < 0.001)	3.1 ± 0.9 (*P* < 0.01)	3.3 ± 1.2 (*P* < 0.01)	5	P1NP; 50%*	TRACP-5b; 5%*	NR
Shimizu et al. (2021) (16)	38	26.2 ± 3.9	12	12.0%*	3.0%*	0.5%*	NR	P1NP; 80%*	TRACP-5b; 5%*	NR
Tominaga et al. (2021) (14)	54	NR	12	8.0%*	NR	1.0%* (NS)	NR	iP1NP; 82.5%	TRACP-5b; 18.6%	No serious
Inage et al. (2021) (17)	35	22.6 ± 14.9	6	4.5 ± 2.6 (*p* = 0.34)	1.0 ± 2.7 (*p* = 0.64)	1.5 ± 1.8 (*p* = 0.62)	NR	P1NP; 186 ± 163.3% (*p* < 0.01)	TRACP-5b; 17.6 ± 22.6% (*p* < 0.01)	No serious
Langdahl et al. (2017) (18) (STRUCTURE)	206	74.4 ± 34.8	12	9.8 (*p* < 0.0001)	3.2 (*p* < 0.0001)	2.9 (*p* < 0.0001)	7 (3%)	P1NP; 20%* (*p* < 0.0001)	CTX; ± 0%*	17 (8%) (serious)
This study	4	68.3 ± 16.9	12	10.2 ± 0.08 (*p* < 0.05)	1.4 ± 0.03 (NS)	−0.8 ± 0.02 (NS)	0	P1NP; 108.9% (NS)	I CTP; 4.2% (NS)	No serious

BMD, bone mineral density; iP1NP, intact type I procollagen N-terminal propeptide; I-CTP, type I collagen cross-linked C-telopeptide; TRAP-5b, isoform 5b of tartrate-resistant acid phosphatase; β-CTX, β-isomer of C-terminal telopeptide of type 1 collagen; NR, not reported; NS, not significant.

* Approximate values inferred from the graphs.

In this study, we revealed that factors affecting the rate of change in lumbar BMD at 12 months were the baseline spine BMD T-score and baseline P1NP values. Moreover, the factor affecting the rate of change in femoral neck BMD at 12 months was the baseline femoral neck BMD T-score, suggesting that romosozumab exerts greater efficacy in patients with severe osteoporosis. Ebina et al. reported that the treatment effect of romosozumab on the BMD increase at 12 months was related to the values of bone formation markers (P1NP) at 1 month for the lumbar spine and the percentage change in bone resorption markers (TRACP-5b) at 1 month for the total hip (13). Tominaga et al. reported that romosozumab showed better efficacy in patients with severe osteoporosis who had lower lumbar BMD, higher TRACP-5b, and higher P1NP levels prior to treatment initiation (14).

Previous studies have shown the incidence of new vertebral fractures during romosozumab treatment ranging from 0.4% to 4% (12, 20). Although a simple comparison cannot be made due to differences in patient backgrounds, one new vertebral fracture (4.8%) was observed in this study. In addition, although the patients in this study were older adults with a median age of 73 years, romosozumab treatment was completed in all patients without any other adverse effects, suggesting that romosozumab could be safely administered to older adult patients. In this study, the median treatment durations before romosozumab administration were 57 months in the denosumab group and 68 months in the bisphosphonate group, which were long enough to consider romosozumab treatment after a long period of antiresorptive drug treatment.

This study had some limitations. The small number of enrolled patients may have reduced the statistical power of the results. Notably, the number of patients who had been pretreated with bisphosphonates was only four, making it impossible to draw a solid conclusion from this study. Obviously, it will be essential to evaluate the effect of romosozumab in a larger number of patients in the future. Further, serum vitamin D levels were not measured in this study because such testing is not reimbursed by the Japanese national health insurance system. Nevertheless, all participants received daily supplementation with calcium (600 mg) and Alfacalcidol (an active vitamin D₃ analog; 1 µg) throughout the study period. In addition, as this was a prospective observational study and not a randomized trial, a placebo treatment was not included, which precluded a direct comparison of treatment effects. In this study, patients whose BMD of the lumbar spine or femoral neck was still below −2.5 SD of the mean value for healthy Japanese women aged 20–44 were recruited. As a result, the median BMD before romosozumab treatment was 0.77 g/cm² at the lumbar spine (T-score: −2.0) and 0.49 g/cm² at the femoral neck (T-score: −2.8). As many previous studies have shown, many elderly women have degenerative changes in their lumbar spine, leading to falsely increased lumbar spine BMDs (21, 22). Thus, Muraki et al. concluded that femoral neck BMD may be more appropriate than lumbar spine BMD for evaluating osteoporosis in elderly women (21). Therefore, we prioritized femoral neck BMD values in this study; however, treatment with romosozumab did not significantly increase the femoral neck BMDs of the participants. Recently, Adami et al. reported a 6-month prospective observational study on postmenopausal women with severe osteoporosis receiving treatment with romosozumab, either alone or in combination with ongoing long-term denosumab, or continuing with ongoing denosumab alone (23). In their study, adding romosozumab to ongoing denosumab resulted in an increase in P1NP and lumbar spine BMD, but not in femoral neck BMD, which is similar to our results. They speculated that patients receiving long-term denosumab may have already developed a higher cortical BMD, potentially limiting the extent of increase attainable with subsequent romosozumab treatment. Further research involving a larger number of patients is needed to clarify the clinical effect of romosozumab following denosumab treatment, with particular focus on the number of fracture events.

In conclusion, we showed that 12 months of romosozumab administration significantly increased the lumbar BMD by 6.1% in postmenopausal women with osteoporosis who were treated with conventional antiresorptive osteoporosis drugs for more than 3 years, followed by a substantial increase in bone turnover markers, including P1NP and I-CTP in this prospective observational study. Romosozumab was effective in postmenopausal patients with osteoporosis who were treated with antiresorptive agents such as bisphosphonates and denosumab for a long time, and that it can be considered as a treatment option for switching therapies.

## Data Availability

The raw data supporting the conclusions of this article will be made available by the authors, without undue reservation.
