# Supplementary material for: Article title efficacy and safety of romosozumab in postmenopausal women with osteoporosis previously treated with antiresorptive drugs: a prospective observational study and literature review
Source: Front Glob Womens Health. 2026 Jul 2;7:1779730. doi: 10.3389/fgwh.2026.1779730 (PMC13373090; doi:10.3389/fgwh.2026.1779730)
Supplement: Supplementary file 1 [file Table1.docx]

**Supplementary Table 1** Multiple regression analysis of percent change in lumbar BMD at 12 months of romosozumab treatment.

| Factor | Coefficient | SE | t | *P* value |
| --- | --- | --- | --- | --- |
| Prior treatment | -0.0006 | 0.0054 | -0.1037 | 0.9214 |
| BMD of the lumbar spine at baseline | -1.3885 | 0.0851 | -16.3235 | <0.0001 |
| BMD of the lumbar spine at 6 months | -0.0187 | 0.1485 | -0.1257 | 0.9049 |
| BMD of the femoral neck at baseline | 0.2144 | 0.1499 | 1.4300 | 0.2121 |
| BMD of the femoral neck at 6 months | 0.0742 | 0.1106 | 0.6702 | 0.5324 |
| BMD of the femoral neck at 12 months | -0.2985 | 0.1925 | -1.5504 | 0.1817 |
| I-CTP value at baseline | -0.0014 | 0.0014 | -0.9890 | 0.3681 |
| Change of　I-CTP level from baseline at 1 months | -0.0045 | 0.0124 | -0.3608 | 0.7330 |
| Change of　I-CTP level from baseline at 6 months | -0.0056 | 0.0127 | -0.4427 | 0.6765 |
| Change of　I-CTP level from baseline at 12 months | -0.0042 | 0.0160 | -0.2656 | 0.8012 |
| P1NP value at baseline | 0.0001 | <0.0001 | 3.6069 | 0.0154 |
| Change of P1NP level from baseline at 1 months | -0.0011 | 0.0028 | -0.4036 | 0.70319 |
| Change of P1NP level from baseline at 6 months | <0.0001 | 0.0005 | 0.1111 | 0.9159 |
| Change of P1NP level from baseline at 12 months | -0.0003 | 0.0008 | -0.3294 | 0.7552 |

SE, standard error; BMD, bone mineral density; P1NP, procollagen type 1 N-terminal propeptide; I-CTP, type I collagen cross-linked C-telopeptide.
