# Supplementary material for: Article title efficacy and safety of romosozumab in postmenopausal women with osteoporosis previously treated with antiresorptive drugs: a prospective observational study and literature review
Source: Front Glob Womens Health. 2026 Jul 2;7:1779730. doi: 10.3389/fgwh.2026.1779730 (PMC13373090; doi:10.3389/fgwh.2026.1779730)
Supplement: Supplementary file 2 [file Table2.docx]

**Supplementary Table 2** Multiple regression analysis of percent change in femoral neck BMD at 12 months of romosozumab treatment.

| Factor | Coefficient | SE | t | *P* value |
| --- | --- | --- | --- | --- |
| Prior treatment | -0.0015 | 0.0027 | -0.5515 | 0.6050 |
| BMD of the lumbar spine at baseline | 0.0357 | 0.0426 | 0.8378 | 0.4403 |
| BMD of the lumbar spine at 6 months | 0.0831 | 0.0744 | 1.1164 | 0.3150 |
| BMD of the lumbar spine at 12 months | -0.1056 | 0.0568 | -1.8583 | 0.1222 |
| BMD of the femoral neck at baseline | -2.0976 | 0.0752 | -27.9078 | <0.0001 |
| BMD of the femoral neck at 6 months | -0.1229 | 0.0555 | -2.2153 | 0.0776 |
| I- CTP value at baseline | -0.0003 | 0.0007 | -0.3605 | 0.7332 |
| Change of　I-CTP level from baseline at 1 months | -0.0099 | 0.0062 | -1.6063 | 0.1691 |
| Change of　I-CTP level from baseline at 6 months | 0.0026 | 0.0064 | 0.4011 | 0.7049 |
| Change of　I-CTP level from baseline at 12 months | -0.0008 | 0.0080 | -0.0941 | 0.9287 |
| P1NP value at baseline | <0.0001 | <0.0001 | 0.8408 | 0.4388 |
| Change of P1NP level from baseline at 1 months | 0.0032 | 0.0014 | 2.2764 | 0.0719 |
| Change of P1NP level from baseline at 6 months | 0.0004 | 0.0003 | 1.5236 | 0.1881 |
| Change of P1NP level from baseline at 12 months | -0.0005 | 0.0004 | -1.3027 | 0.2495 |

SE, standard error; BMD, bone mineral density; P1NP, procollagen type 1 N-terminal propeptide; I-CTP, type I collagen cross-linked C-telopeptide.
