# Supplementary figures and images for: Article title efficacy and safety of romosozumab in postmenopausal women with osteoporosis previously treated with antiresorptive drugs: a prospective observational study and literature review
Source: Front Glob Womens Health. 2026 Jul 2;7:1779730. doi: 10.3389/fgwh.2026.1779730 (PMC13373090; doi:10.3389/fgwh.2026.1779730)

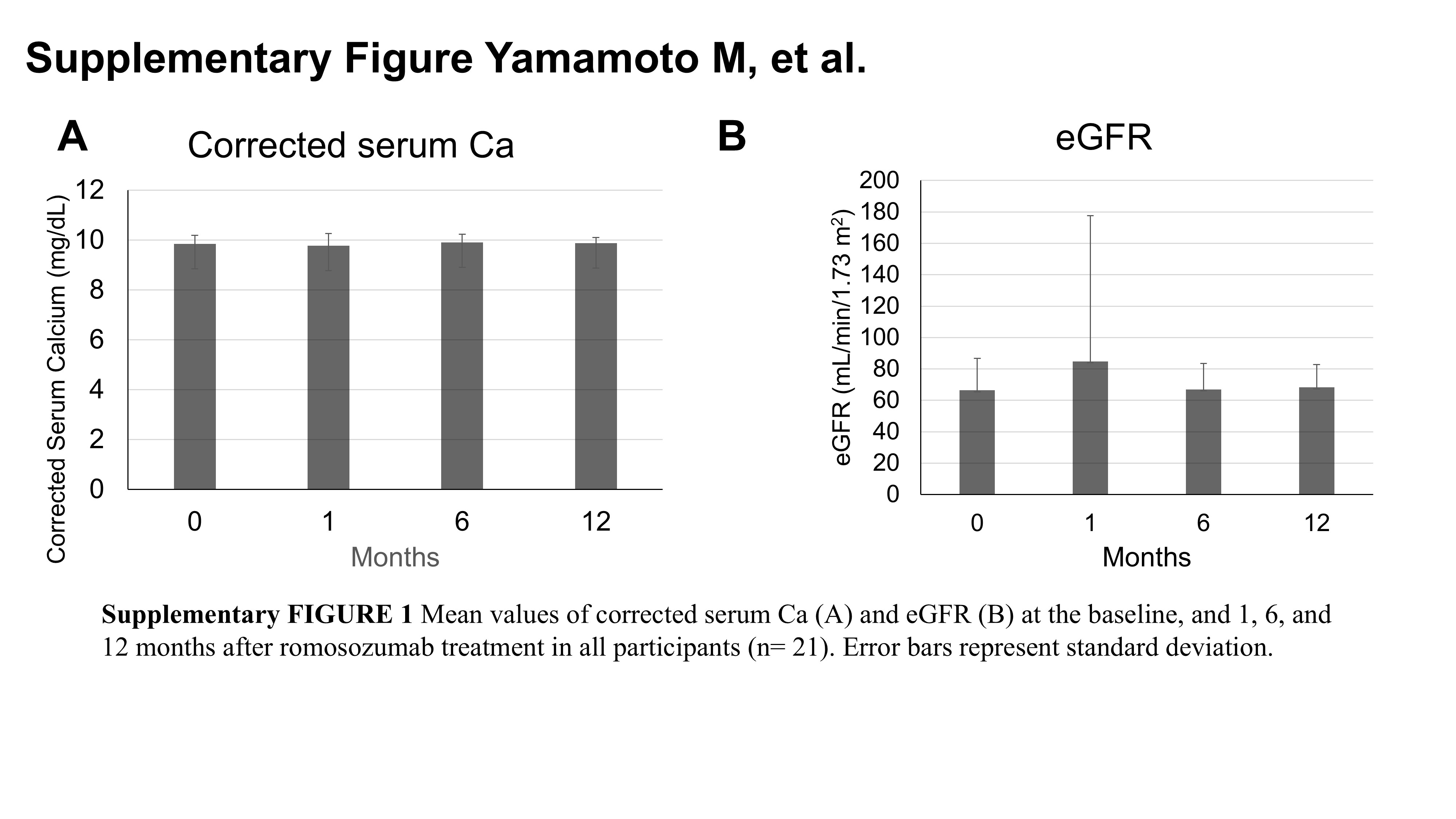

Supplement: Supplementary file 3 [file Image1.jpeg]
